# The prognostic potential of circulating biomarkers for sarcoma patients with pleural dissemination

**DOI:** 10.3389/pore.2025.1612133

**Published:** 2025-09-22

**Authors:** Hannah Schwab, Stéphane Collaud, Sebastian Bauer, Uta Dirksen, Dirk Theegarten, Fabian Doerr, Konstantinos Grapatsas, Natalie Baldes, Clemens Aigner, Servet Bölükbas, Balazs Hegedüs

**Affiliations:** ^1^ Department of Thoracic Surgery, University Medicine Essen – Ruhrlandklinik, Essen, Germany; ^2^ Department of Thoracic Surgery, Cologne Merheim Hospital, University of Witten/Herdecke, Cologne, Germany; ^3^ Department of Oncology, University Medicine Essen, Essen, Germany; ^4^ Department of Pediatric Hematology and Oncology, University Medicine Essen, Essen, Germany; ^5^ Institute of Pathology, University Medicine Essen, Essen, Germany; ^6^ Department of Thoracic Surgery, Medical University of Vienna, Vienna, Austria

**Keywords:** sarcoma, pleural effusion, lactate dehydrogenase, CRP, prognosis

## Abstract

Sarcomas are a heterogeneous group of rare and aggressive malignancies and have a propensity to metastasize to the thoracic cavity. While sarcoma lung metastasectomy is an established modality, only scarce information is available about potential prognostic factors for sarcoma patients with pleural dissemination. Accordingly, all consecutive sarcoma patients treated at our thoracic surgery department between 2010 and 2023 with pleural sarcomatosis and/or malignant pleural effusion were retrospectively analyzed. Preoperative circulating biomarker values were collected at the time of first pleural involvement. Overall survival was calculated from the first sarcoma diagnosis as well as from the first diagnosis of pleural dissemination. 98 patients (42 female) were included in the cohort with a median age of 54.6 years (range: 15.9–84.3 years) at the time of pleural involvement. 77 patients had soft tissue sarcoma, while 21 patients had primary sarcoma in the bone including 4 chondrosarcoma. Among the 19 different sarcoma types, synovial sarcoma (13%), liposarcoma (11%), undifferentiated pleomorphic sarcoma (11%), Ewing (like) sarcoma (10%) and leiomyosarcoma (9%) were the most frequent. Pleural dissemination was mostly metachronous, while only 7 cases were synchronous. The median pleural dissemination-free interval was 17.1 months after sarcoma diagnosis. The median overall survival after pleural dissemination was 12 months. WBC values outside the normal range had no significant impact on overall survival. High LDH (>250 U/L) and CRP (>1 mg/dL) conferred significantly lower overall survival (8.6 months vs. 19.1 months (p < 0.0001) and 4.9 months vs. 29 months (p < 0.0001), respectively). Albumin alone showed no prognostic impact, however, the modified Glasgow prognostic score (0, 1, and 2) was a strong prognosticator (20.4 vs. 8.6 vs. 1.7 months (p < 0.0001). In a multivariable analysis, CRP remained a significant prognostic factor. In conclusion, routine circulating biomarkers carry prognostic information for sarcoma patients with pleural dissemination and should be considered for risk stratification and personalized therapeutic decisions.

## Introduction

Sarcomas are a rare, aggressive and heterogeneous group of malignancies including more than 100 different types of tumors of soft tissue and bones [1]. The most common histological subtypes in adults, classified by their tissue of origin, are liposarcoma, leiomyosarcoma and undifferentiated pleomorphic sarcomas. Sarcomas are responsible for 1% of all cancer in adult patients and can arise in any part of the body at any age [2]. They most commonly metastasize to the lungs [3]. Due to the large number of subtypes of sarcomas, the prognosis and treatment options are also widely heterogeneous. Therefore, an interdisciplinary individual treatment concept should be established for each patient [2].

Malignant tumors can oftentimes spread to the pleura resulting in malignant pleural effusion and in pleural carcinosis or sarcomatosis. Malignant pleural effusion (MPE) is associated with shorter overall survival in the overwhelming majority of malignancies, even though the concrete mechanism of a MPE resulting in poor prognosis remains still unclear [4]. Interestingly, even non-malignant pleural effusion can have an impact on the prognosis of cancer patients [4].

Sarcomas can also disseminate to the pleura, however, they are responsible for a small fraction of malignant effusions [5]. Among sarcomas rhabdomyosarcoma, osteosarcoma and Ewing sarcoma are most often found in pleural or cerebrospinal fluids [6]. Nevertheless, there is only limited information available about the prognostic impact of pleural dissemination in sarcoma [6, 7].

Several circulating biomarkers have already been investigated in a variety of sarcoma subtypes. Most studies are focusing on soft tissue sarcomas or specific subtypes of sarcoma. C-reactive protein (CRP), serum albumin, lactate dehydrogenase (LDH) and modified Glasgow prognostic score has already been identified as prognostic marker for soft tissue sarcomas [8–12]. There are a limited number of biomarker studies focusing on metastatic sarcomas agnostic of the primary sarcoma entities [13].

Altogether, the prognostic potential of circulating biomarkers in sarcoma patients with pleural dissemination has not yet been studied. For this very reason, we investigated potential clinical prognostic factors and serum biomarkers for sarcoma patients with pleural involvement.

## Materials and methods

The study cohort includes 98 sarcoma patients who underwent a surgical procedure at Department of Thoracic Surgery and Thoracic Endoscopy, University Medicine, Essen, Germany between 2010 and 2023 with pleural dissemination of their primary sarcoma. All clinical data were retrospectively collected from the clinical electronic documentation system.

The criteria of pleural involvement include metastases in the pleura and/or malignant pleural effusion. Pleural involvement was diagnosed with histological and cytological samples in 81 (83%) patients. 17 (17%) patients had a clinical diagnosis via CT, X-ray or sonography.

Pleurabiopsies or surgical resection specimens were formalin-fixed and paraffin-embedded. If the histological and/or molecular pathological analysis of the primary sarcoma was performed in our pathology then pleurasarcomatosis was established based on the histopathological correspondence to the primary sarcoma histology. In case of uncertainties or external pathological reports of the primary tumor, sarcoma subtype specific immunohistochemistry panel stainings were performed (e.g., epitheloid sarcoma panel, round cell sarcoma panel).

If molecular pathological analysis was clinically indicated DNA and/or RNA was isolated from the FFPE sections. Fusion analysis was performed by using the Archer FusionPlex Sarcoma Panel and sequencing was performed at the MiSeq platform (Illumina, San Diego, CA, United States). For DNA mutation analysis, NGS libraries were created using a customized QIAseq targeted DNA panel (Qiagen). The pooled library was sequenced on MiSeq (Illumina) platform.

Routine FISH procedure was performed using the ZytoLight^®^ SPEC EWSR1 Dual Color Break Apart Probe or EWSR1/FLI1 TriCheck™ Probe (ZytoVision, Bremerhaven, Germany) for suspected EWSR1 associated sarcoma cases.

Pleural effusion cytology was first performed on cytospin preparations. If a sufficient amount of cells was present in the effusion, cell blocks were prepared and hematoxylin&eosin or immunohistochemical stainings were performed to identify sarcoma cells in the effusions.

All initial therapeutic decisions were made after presenting the case in the certified multidisciplinary sarcoma tumor board.

The levels of circulating biomarkers were taken at the time of diagnosis of first pleural involvement. As most patients had a surgical procedure, preoperative data was collected.

The samples were analyzed in the central laboratory of our clinic following the standards of the International Federation of Clinical Chemistry and Laboratory Medicine (IFCC). Serum CRP was evaluated as a categorical variable as CRP values below 0.5 were not metrically documented. LDH was analyzed as a metric and a categorical variable as well. CRP and albumin were also combined in the modified Glasgow Prognostic Score (mGPS). Patients with a normal albumin or hypoalbuminemia (<3.5 g/dL) and CRP <1 mg/dL are classified with mGPS 0, patients with an increased CRP (>1 mg/dL) and a normal albumin or hypoalbuminemia (<3.5 g/dL) were classified with mGPS 1 and patients with both increased CRP and hypoalbuminemia were classified with mGPS 2 [10]. The Ethic Committee of the Medical Faculty of University Duisburg-Essen approved the study (approval number: 17-7775-BO) and waived the requirement to obtain informed consent for this retrospective study covering two decades.

### Statistical analysis

To identify potential prognostic markers, we analyzed overall survival using the Kaplan-Meier method. Regarding the clinical factors and circulating biomarkers we compared different levels and their impact on the overall survival using the log-rank and Gehan-Breslow-Wilcoxon Test.

Overall survival was calculated from two different points of time. On the one hand, from the primary diagnosis of sarcoma and, on the other hand, from the date of first pleural involvement. The time of first pleural involvement was defined as the first evidence of a sarcomatosis or effusion in pathological or clinical examinations.

## Results

Fifty-six (57%) of the patients were male, 42 (43%) female. The median age of the patients at primary diagnosis was 53.2 years (5.4–83.9 years), while 24 (24%) were under 30, 47 (48%) were between 30 and 60 and 27 (28%) were older than 60 at the time of diagnosis. The median age at the pleural involvement was 54.6 years (range: 15.86–84.3 years). The pleural involvement free interval ranged from 0–248.6 months with a median of 17.1 months. Most of the patients developed the pleural involvement metachronous (>3 months) to their primary diagnosis (91) while only 7 patients had synchronous pleural involvement (diagnosis within 3 months of the primary diagnosis). The cohort included 19 different sarcoma subtypes (Supplementary Table S1). Seventy-seven patients had soft tissue sarcoma (79%) while 21 patients had primary sarcoma in the bone (21%). Similarly, the localization of the primary tumor was also very diverse. Thirty-seven patients were diagnosed with a tumor at their lower extremity, 23 at their upper extremity, 16 at their abdomen or pelvis, 14 in their thoracic region and 7 at their head/neck. The original localization of one patient’s tumor was not identified.

**FIGURE 1:**
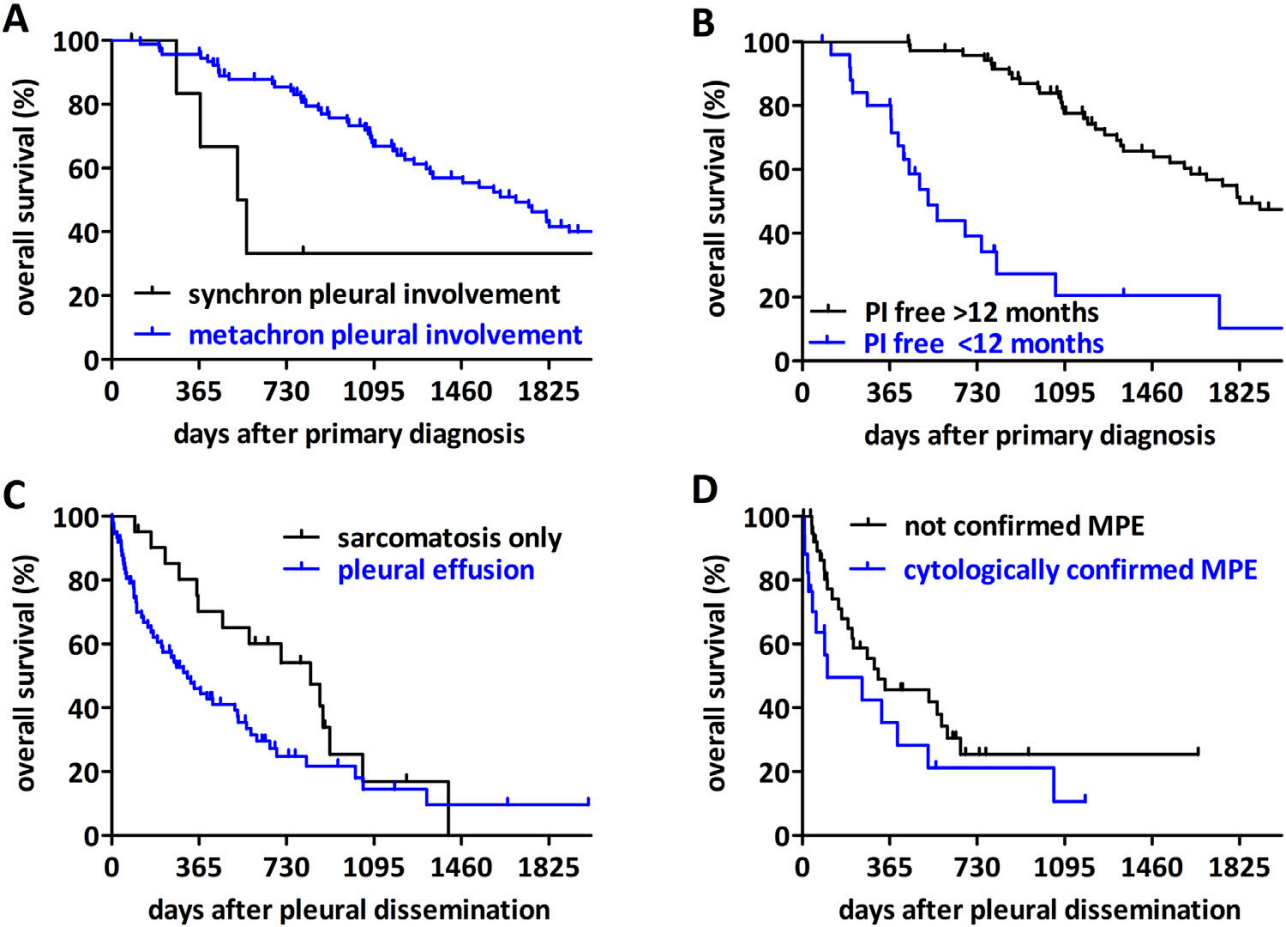
The impact of clinical factors on survival of sarcoma patients with pleural involvement (PI). **(A)** Patients with synchronous pleural involvement (<3 months from primary diagnosis) have shorter overall survival after diagnosis compared to metachronous cases (p = 0.0098). **(B)** Patients with a pleural involvement-free interval shorter than 12 months have a worse overall survival (p < 0.0001). **(C)** Patients with pleural sarcomatosis without effusion have a longer overall survival after diagnosis of pleural involvement than patients with pleural effusion (p = 0.0295). **(D)** Cytologically verified malignant pleural effusion confers a shorter overall survival than patients with pleural effusion where no malignant cells were identified (p = 0.0451).

The median survival of all patients after primary diagnosis was 137 months (2–271 months), the median survival after diagnosis of pleural involvement was 12 months (0–65 months). Age, sex and primary tumor grade had no significant impact on median overall survival (Table 1). Early pleural dissemination–either synchronous or within 1 year of the primary diagnosis – conferred worse survival after primary diagnosis (Figures 1A,B).

**TABLE 1:** The impact of age, sex and primary tumor grade on median overall survival.

Variable	Value	# Of cases (%) N = 98	Survival after primary diagnosis (months)	p-value	Survival after pleural involvement (months)	p-value
sex	Male	56 (57)	50.4	0.529	17.4	0.221
	Female	42 (43)	53.3		9.8	
age at diagnosis	<30 ys	24 (24)	80.1	0.279	18.8	0.257
	30–60 ys	47 (48)	52.4		11.8	
	>60 ys	27 (28)	40.2		10.4	
age at pleural involvement	<30 ys	19 (19)	44.0	0.800	18.5	0.335
	30–60 ys	45 (46)	52.4		13.1	
	>60 ys	34 (35)	53.3		8.6	
grade of primary tumor (NA = 31)	grade 1	2 (3)	60.5	0.975	29.5	0.244
	grade 2	18 (27)	55.5		3	
	grade 3	47 (70)	39		11	

The pleural involvement was verified by histology or cytology in 81 patients and 17 patients had only clinical diagnosis. There was no difference in the overall survival after pleural dissemination between these two subcohorts (Supplementary Figure S1). Seventy-seven patients had pleural effusion and 21 patients presented only pleural sarcomatosis. The majority (55) of the patients with an effusion had a one-side effusion, while 10 of them were affected on both lung sides. For 12 patients laterality was not available. In our cohort, patients with pleural effusion had a significantly shorter overall survival after primary diagnosis compared to patients with sarcomatosis only (Figure 1C). The analysis of the effusion cases with definitive cytological report (61 patients), exhibited a modest but significant difference between patients with a cytological confirmed malignant pleura effusion and patients where no malignant cells were identified in the effusion (Figure 1D).

**FIGURE 2:**
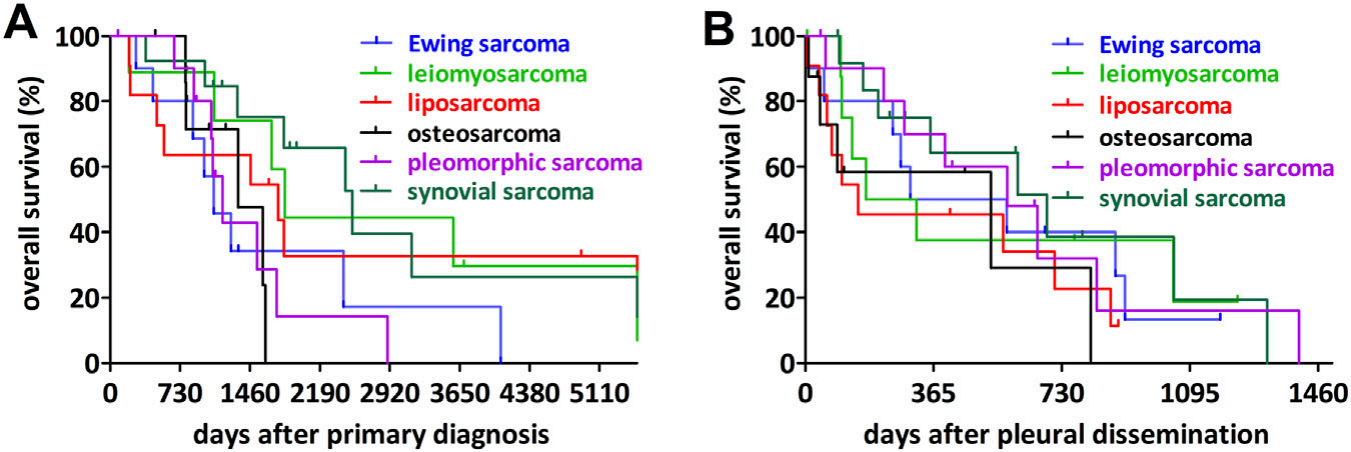
Impact of the most frequent sarcoma entities on overall survival. **(A)** Synovial sarcoma had the longest overall survival after primary diagnosis while osteosarcoma, undifferentiated pleomorphic sarcoma and Ewing sarcoma associated with the shortest overall survival. **(B)** Analyzing the overall survival after diagnosis of the pleural dissemination no significant differences could be detected.

The cohort included 19 different sarcoma entities. Accordingly, the median overall survival and the differences between the sarcoma subtypes could only be compared for the most frequent ones (Table 2; Figure 2).

**TABLE 2:** Median overall survival in the different sarcoma entities.

Sarcoma subtype	# Of cases (%) N = 98	Survival after primary diagnosis (months)	Survival after pleural involvement (months)
synovial sarcoma	13 (13)	79	16
liposarcoma	11 (11)	85	12
pleomorphic sarcoma (UPS)	11 (11)	39	19
Ewing (like) sarcoma^a^	10 (10)	45	16
leiomyosarcoma	9 (9)	72	14
osteosarcoma	8 (8)	40	9
others	36 (36)	NA	NA

^a^ This group includes one Ewing-like sarcoma case with a CIC-DUX4, fusion.

In the pairwise comparison, osteosarcoma was associated with significantly shorter overall survival after the primary diagnosis when compared to synovial sarcoma or leiomyosarcoma and Ewing sarcoma showed a similar tendency, however not statistically significant. In contrast, we found no significant differences between the sarcoma entities in terms of overall survival after the pleural dissemination.

**FIGURE 3:**
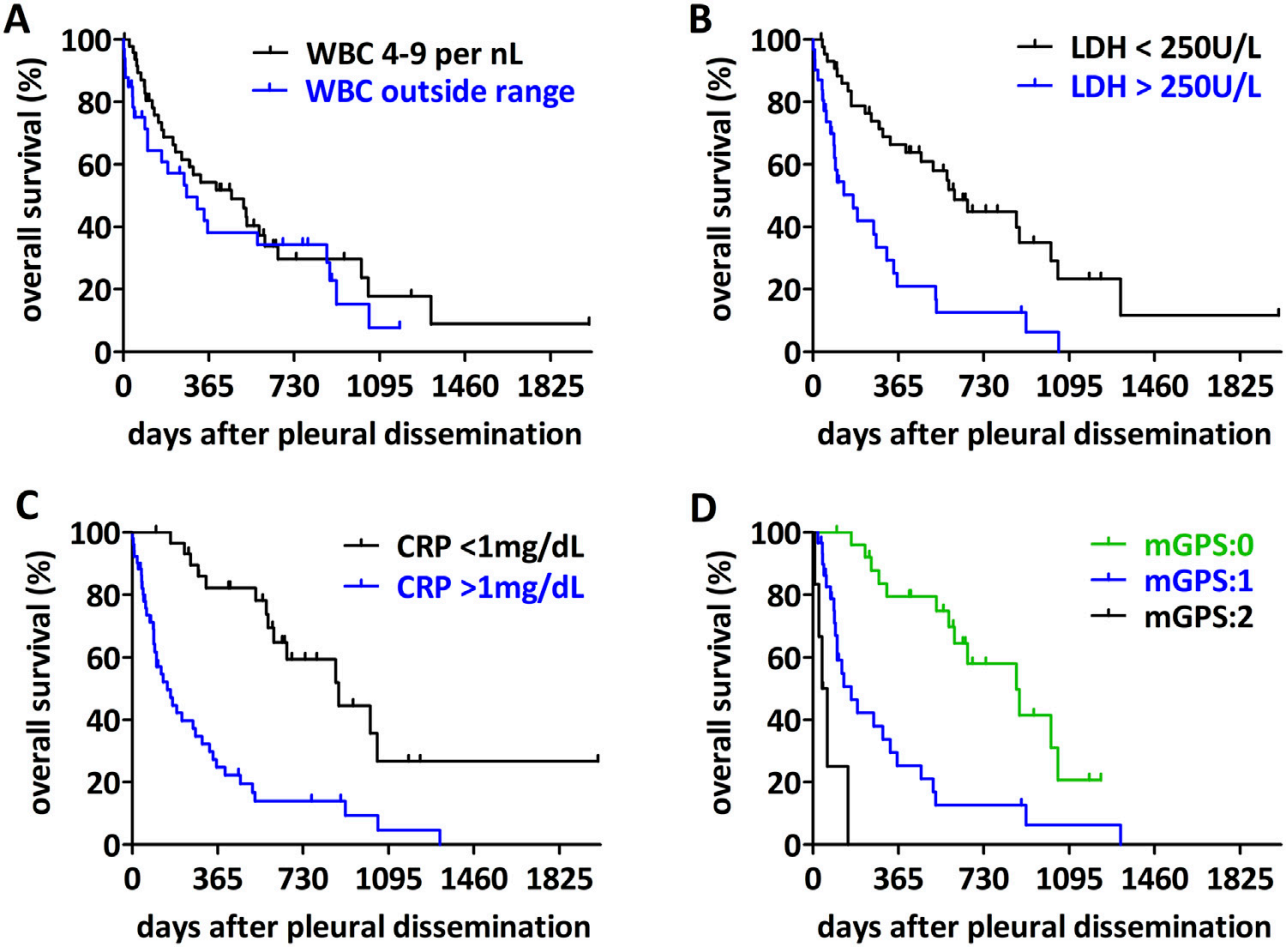
Impact of circulating biomarkers on overall survival of sarcoma patients with pleural dissemination. **(A)** For patients with a WBC count outside the normal range (4–9/nL) no difference in the overall survival could be detected (p = 0.3251). **(B)** Patients with a high LDH-level (>250 U/L) have a worse overall survival (p < 0.0001). **(C)** Patients with a high CRP-level (>1 mg/dL) have a shorter overall survival (p < 0.0001). **(D)** Analyzing the impact of the mGPS-score, patients with a higher score have a worse overall survival (p < 0.0001).

Regarding the impact of circulating biomarkers, we analyzed the median overall survival from the time of pleural dissemination when the biomarkers values were collected (Figure 3; Table 3). There was no significant difference between patients with white blood cell count within the normal range (4-9/nL) or outside the normal range (Figure 3A, p = 0.3251). However, patients with higher LDH levels have a significantly poorer outcome compared to patients with low LDH level (Figure 3B, cut-off 250 U/L, p < 0.0001). CRP also had a significant impact on the median overall survival (Figure 3C, p < 0.0001). Albumin levels alone had no significant influence on overall survival (p = 0.4412, Supplementary Figure S1). The modified Glasgow prognostic score identified three significantly different prognostic groups (Figure 3D, p < 0.0001). Finally, in a multivariable analysis of the significant serum biomarkers LDH and CRP together with the type of pleura involvement, CRP remained a significant prognostic factor (Table 3).

**TABLE 3:** Median overall survival after pleural involvement.

				Univariable analysis		Multivariable analysis	
Variable	Category	# (%)	Survival (month)	HR (95% CI)	p-value	HR (95% CI)	p-value
CRP NA:14	low	30 (36)	29.0	3.99 (2.29–6.96)	**<0.0001**	0.30 (0.15–0.63)	**0.001**
	high	54 (64)	4.9				
LDH NA:21	low (<250)	45 (58)	19.1	0.28 (0.15–0.53)	**<0.0001**	0.65 (0.34–1.24)	0.188
	high (>250)	32 (42)	8.6				
Type	sarcomatosis only	21 (21)	27.6	0.62 (0.37–1.04)	**0.0086**	0.58 (0.29–1.20)	0.141
	effusion	77 (79)	10.5				
MPE NA:16	not confirmed	17 (28)	10.4	1.77 (0.83–3.75)	**0.0451**		
	confirmed	44 (72)	3.4				
WBC NA:14	4–9/nL	51 (61)	15.2	0.76 (0.43–1.32)	0.1649		
	out of range	33 (39)	8.9				
mGPS NA:28	0	26 (37)	20.4		**<0.0001**		
	1	32 (46)	8.6	0.23 (0.11–0.47)			
	2	12 (17)	1.7	0.0002 (0.0001–0.0027)			

Bold p‐values are significant.

## Discussion

Pleural dissemination in sarcomas is a very uncommon course of the disease. Due to the rare occurrence, negligible information considering this clinical condition is available and prognostic factors are barely studied in this patient population. In our retrospective analysis of sarcoma patients with pleural involvement, we analyzed the influence of clinical factors as well as circulating biomarkers on the outcome. Regarding the impact of pleural effusion in cancer patients, pleural dissemination including malignant pleural effusion and pleural metastasis is a negative prognostic factor and means lower life expectancy in most cancer types, while the exact impact of pleural effusion remains unclear [4, 14]. Porcel et al. calculated a median survival for lung cancer patients with a malignant pleural effusion of 5.5 months. They also outlined the significant impact of even a small pleural effusion in comparison to patients without pleural effusion (7.5 vs. 12.7 months). Also, any other kind of pleural involvement than effusion led to lower overall survival in lung cancer patients. Other studies report an overall survival of cancer patients with malignant pleural effusion of 4–9 months [15], 4–7 months [4] or 3–12 months [16]. The median survival after pleural involvement in our study cohort was reduced to 12 months. The majority of effusions occurred unilaterally emphasizing the interpretation of the effusion as a cancer-related complication. Patients who are already diagnosed with a pleural involvement or develop it less than 12 months after primary diagnosis have a lower life expectancy. This was already described for lung cancer patients earlier [17]. The impact of the pleural involvement free interval for sarcoma patients is rarely studied. Di Carlo et al. observed that a pleural involvement at the time of diagnosis is a poor prognostic factor for children with rhabdomyosarcoma. But they also assert that there is no published data on the role of tumor-associated effusion in patients with rhabdomyosarcoma [7]. This observation is in line with our finding that sarcomas with synchronous pleural dissemination have a particularly poor prognosis.

Not only the pleura involvement free interval has an impact on the overall survival of our study cohort, but also the type of pleural involvement. Patients with an effusion showed shorter overall survival than patients with sarcomatosis only. Sharma et al. described this also for cancer patients in general [16]. Reviewing the literature, an effusion without detected malignant cells but with confirmed malignancy in the pleural cavity is defined as paramalignant effusion [14]. Interestingly, we found a modest but significant difference in overall survival between patients with cytologically confirmed or not confirmed malignant pleural effusion. Of note, all patients in our cohort presented an involvement of the pleural cavity. Nevertheless, some of the non-malignant effusions might be the result of other comorbidities and not directly due to the sarcoma dissemination and may contribute to this difference. In line with the pan-cancer metaanalysis by Yang et al, we found that pleural effusion had an impact on overall survival, regardless whether it is confirmed malignant or not [4].

There are several studies examining the impact of CRP, albumin and LDH as prognostic factors for patients with sarcoma, mostly for specific sarcoma subtypes. LDH is described as a prognostic factor for patients with high-grade sarcoma [18] and for Ewing sarcoma [19, 20]. Li et al. describe high serum levels of LDH as a prognostic factor for sarcoma patients, indicating low OS and 5-year disease free survival [21]. Furthermore, LDH is declared as prognostic factor in soft tissue sarcoma in general [22]. In our study cohort, LDH was a significant factor for poor overall survival, if the serum level was higher than 250 U/L at the time of pleural involvement diagnosis.

A number of studies investigated CRP as prognostic factor for soft tissue sarcoma or bone sarcoma patients [23, 24]. Preoperative elevated CRP level was a negative prognostic factor for soft tissue sarcoma patients who underwent surgery in curative intention [9, 12]. CRP showed prognostic impact in soft tissue sarcoma with metastases at initial presentation [25] but also before any treatment [5, 11]. CRP was identified as a prognostic factor for certain bone sarcoma subtypes including in Ewing sarcoma [23]. However, our study is the first to demonstrate the prognostic impact of serum CRP at the time of pleural dissemination. Importantly, CRP remained a significant prognostic factor in multivariable analysis.

Albumin was identified as independent prognostic factors for STS patients with metastasis at time of diagnosis previously [25]. While albumin alone had no prognostic impact in our cohort, we did find a significant impact of mGPS on overall survival. There are several studies studying mGPS as a prognostic factor in sarcomas. Spence et al. demonstrated that an elevated mGPS correlates with a poor prognosis of localized STS [26]. mGPS is described as an independent predictor for recurrence-free and cancer-specific survival in patients with soft tissue and bone sarcoma who underwent surgery [27] and as a significant prognostic factor for patients with retroperitoneal soft tissue sarcoma [28]. Our examination is the first to show the use of mGPS as a prognostic factor for sarcoma patients with pleural involvement.

White blood cell count had no association with prognosis in our metastatic sarcoma cohort. In localized bone and soft tissue sarcoma, abnormally low absolute lymphocyte count at diagnosis is associated with higher mortality [29].

Our study suffers certain limitations through its retrospective design. All patients are collected from a single thoracic surgery center which might induce a selection bias. Patients with pleural involvement but without indication for a surgical procedure could not be included in our cohort. We also acknowledge that in the rapidly evolving landscape of sarcoma pathology some of the entities included are no longer considered *bona fide* sarcoma or their categorization has changed due to the identification of novel molecular subgroups. Furthermore, there might be certain comorbidities that might impact overall survival but could not be systematically assessed in our database.

In summary, we demonstrated that a sarcoma pleural involvement is associated with a poor outcome. If the pleural involvement occurs soon after the primary diagnosis, it lowers the overall survival. Our study indicates that LDH, CRP and mGPS can be used as prognostic factors for patients with sarcoma pleural dissemination at time of diagnosis of pleural involvement and should be considered for personalized therapeutic decisions.

## Data Availability

The raw data supporting the conclusions of this article will be made available by the authors, without undue reservation.
