# Supplementary material for: The prognostic potential of circulating biomarkers for sarcoma patients with pleural dissemination
Source: Pathol Oncol Res. 2025 Sep 22;31:1612133. doi: 10.3389/pore.2025.1612133 (PMC12497659; doi:10.3389/pore.2025.1612133)

Supplementary Table 1. Sarcoma subtypes

Sarcoma Subtype	Number (%)	median survival after primary diagnosis (months)	median survival after pleural involvement (months)
Alveolar soft tissue sarcoma	1 (1)	192	54
Angiosarcoma	2 (2)	25	15
Carcinosarcoma #	3 (3)	34	11
Chondrosarcoma	4 (4)	30	13
Epitheloid sarcoma	5 (5)	65	15
Ewing (like) sarcoma *	10 (10)	45	16
Fibrosarcoma	3 (3)	53	14
Malignant granular cell tumor	1 (1)	14	4
Clear cell sarcoma	4 (4)	55	7
Leiomyosarcoma	9 (9)	72	14
Liposarcoma	11 (11)	85	12
Malignant mesenchymoma	1 (1)	24	11
Myxofibrosarcoma	3 (3)	31	0.5
Osteosarcoma	8 (8)	40	9
Pleomorphic sarcoma (UPS)	11 (11)	39	19
Pulmonary artery sarcoma	1 (1)	67	65
Rhabdomyosarcoma	2 (2)	21	7
Spindle cell sarcoma	6 (6)	95	7
Synovial sarcoma	13 (13)	79	16

# At the time of primary diagnosis carcinosarcoma was still classified by WHO as mixed epithelial and mesenchymal tumor.

* This group includes one Ewing-like sarcoma case with a CIC-DUX4 fusion.

Supplementary table 2: Statistical analysis of overall survival for all variables

Variables		Total n=98 (%)	median survival after primary diagnosis months	P value logrank	P value Gehan-Breslow-Wilcoxon	HR (95% CI)	median survival after pleural Involvement	P value logrank	P value Gehan-Breslow-Wilcoxon	HR (95% CI)
Sex	Male	56 (57)	50.43	0.9389	0.5285	0.98 (0.60 – 1.60)	17.4	0.1694	0.2212	0.70 (0.43 - 1.16)
	Female	42 (43)	53.33				9.8			
Age at diagnosis	< 30 years	24 (24.5)	80.1	0.2785			18.8	0.2572		
	30 – 60 years	47 (48.0)	52.4				11.8			
	> 60 years	27 (27.6)	40.2				10.4			
Age at pleural involvement	< 30 years	19 (19.4)	44.0	0.7988			18.5	0.3347		
	30 – 60 years	45 (45.9)	52.44				13.1			
	> 60 years	34 (34.7)	53.33				8.6			
Leukocytes	4 – 9	51 (52.0)	52.4	0.8655	0.9729	1.05 (0.61 - 1.81)	15.2	0.3251	0.1649	0.75 (0.43 - 1.32)
	Outside regular range	33 (33.7)	55.5				8.9			
	NA	14 (14.3)								
CRP	Low (<1)	30 (30.6)	83.2	**0.0062**	**0.0018**	0.48 (0.27 – 0.81)	29.0	**< 0.0001**	**< 0.0001**	3.99 (2.29 - 6.96)
	High (> 1	54 (55.1)	38.6				4.9			
	NA	14 (14.3)								
Albumin	Low	52 (53.1)	52.4	0.4412	0.6848	1.39 (0.60 - 3.18)	10.4	0.1979	0.1292	1.66 (0.77 - 3.58)
	High	11 (11.2)	92.1				33.4			
	NA	35 (35.7)								
mGPS	0	26 (26.5)	83.2	**0.0297**			20.4	**< 0.0001**		
	1	32 (32.7)	40.2				8.6			
	2	12 (12.2)	16.1				1.7			
	NA	28 (28.6)								
LDH	Low (<250)	45 (46.0)	19.1	0.2084	**0.0401**	0.67 (0.36 - 1.25)	19.1	**< 0.0001**	**< 0.0001**	0.28 (0.15 - 0.53)
	High (>250)	32 (32.7)	8.9				8.6			
	NA	21 (21.4)								
Type of pleural involvement	Sarcomatosis	21 (21.43)	80.81	**0.0105**			27.6	**0.0295**		
	Effusion	29 (29.6)	57.67				11.3			
	Sarcomatosis + Effusion	48 (49)	38.6				9.8			
Timing of pleural involvement	Synchronus	7 (7.1)	17.88	0,0513	**0.0098**	4.84 (0.99 - 23.66)	17.9	0.3034	0.2775	1.47 (1.11 - 1.83)
	Metachronus	91 (92.9)	57.2				12.7			
PI-free interval	< 12 month	26 (26.5)	17.3	**< 0.0001**	**< 0.0001**	18.31 (7.20 - 46.54)	12.2	0.8484	0.8601	0.95 (0.55 - 1.63)
	>12 month	72 (73.5)	60.0				16.9			
Malignant pleural effusion	Cytological non confirmed	17 (17.3)	43.7	0.5276	0.8455	1.26 (0.62 to 2.56)	10.4	0.1423	**0.0451**	1.77 (0.83 - 3.78)
	Cytological confirmed	44 (44.9)	43.2				3.4			
	NA	16 (16.3)								

Supplementary Figure 1. Impact of pathological verification of pleural dissemination on overall survival.

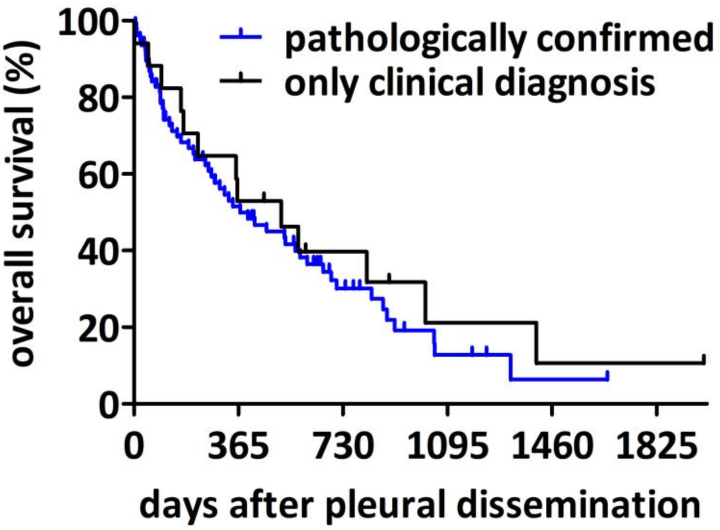

Supplementary Figure 2. Impact of albumin on overall survival

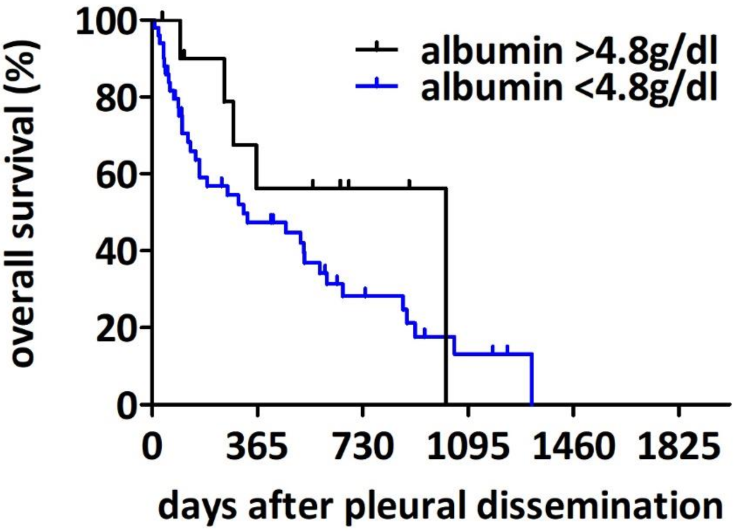

Supplement: Supplementary file 1 [file DataSheet1.pdf]
